# Negotiating the Gerontological Uncanny and Passing Tactics for Aging in Place

**DOI:** 10.1093/geroni/igaa057.2581

**Published:** 2020-12-16

**Authors:** Jarmin Yeh, Pat Fox, David Vlahov, Howard Pinderhughes

**Affiliations:** 1 University of California, San Francisco, San Francisco, California, United States; 2 Yale University, Orange, Connecticut, United States

## Abstract

Insisting people are equally worthy of aging in place is a radical but challenging idea. Scenes of homelessness texture the contemporary city, manifesting broader issues of inequality. This qualitative study explored material with twenty-two housed and unhoused San Franciscans born in 1950 or earlier, who participated in semi-structured in-depth interviews and chronicled their everyday lives using disposable cameras. Theoretical work on social and spatial practices was drawn upon to help understand their aging in place experiences. In interpreting themes, informants elucidated a moving tension between the daily interiority of identity and the negotiation of a changing environment that produces a sensation characterized as the uncanny. The vicissitudes of life and precariousness of their positionalities exposed tactics for “passing” as creative forms of resistance to their expulsion from society. Utilizing visual methods helped reveal assumptions and contributed to gerontological discourse and critical theorizations about aging in place inequalities. Part of a symposium sponsored by the Qualitative Research Interest Group.

